# Developing a competency profile for newly graduated registered nurses in South Africa

**DOI:** 10.1186/s12912-020-00453-7

**Published:** 2020-07-16

**Authors:** Gerhard Hendrik Rabie, Tinda Rabie, Monica Dinkelmann

**Affiliations:** 1 School for Industrial Psychology and Human Resource Management, WorkWell, North-West University, Potchefstroom Campus, Potchefstroom, South Africa; 2 School of Nursing Science, NuMIQ Focus Area, North-West University, Campus, 11 Hoffman Street, Potchefstroom, South Africa

**Keywords:** Competency profile, Knowledge, Skills, Attitudes, Registered nurses, Quality patient care

## Abstract

**Background:**

Newly graduated registered nurses experience various challenges when entering the clinical practice environment. Typical challenges include lack of specific knowledge, skills and attitude competencies which is aggravated further by factors such as transition problems, workloads, lack of confidence and independence which potentially causes poor quality care. The aim of the study was to develop a competency profile for newly graudated registered nures, based on the perceptions of both nurse educators and final-year nursing students regarding the knowledge, skills and attitudes needed to deliver quality patient care in South Africa.

**Methods:**

A qualitative descriptive design was used. Semi-structured individual interviews were conducted with 42 participants consisting of 23 nurse educators and 19 final-year nursing students at three nursing education institutions. The interviews were guided by an interview guide that examined three predetermined themes: knowledge, skills and attitudes as competencies to deliver quality patient care. Data were processed using thematic analysis.

**Results:**

The predetermined theme *knowledge,* was broken down into themes: theoretical knowledge, holistic care, cultural diversity and code of conduct, with its relating sub-themes. The predetermined theme *skills* delivered the following themes: interpersonal, management, administrative, practical and personal skills with its sub-themes. *Attitudes* unpacked into the following themes: being positive, caring, humble, friendly, empathetic, life-long learning, going the extra mile, compassionate, having passion, approachable, sensitive, helpful, and non-judgemental.

**Conclusions:**

Rich, in-depth knowledge, skills and attitudes were identified to develop a competency profile that may assist newly graduated registered nurses when entering the clinical practice environment to deliver quality patient care.

## Background

Competency challenges that newly graduated registered nurses (NGRNs) experience when entering the clinical practice environment have been widely researched. Challenges identified in various studies on NGRNs include transition problems, overwhelming workloads, poor organisation for patient care and inadequate responses to patients’ problems caused by lack of competence [1]. South African (SA) studies indicate that NGRNs have a lack of knowledge and skills, insufficient record keeping and are not following protocols [2], which again could be due to competency issues. Certain NGRNs show a lack of independence and confidence, as well as feelings of incompetence [3, 4]. Further challenges were found to be: feeling overwhelmed and struggling with the transition from student to NGRN [5], insufficient practical experience as well as deficiencies and adequacies in their professional role [3].

The SA Department of Health notes that certain NGRNs may experience role conflict, which confirmed findings from literature that such nurses experience incompetence to fulfil their role as NGRNs [6]. The mentioned nurses thus feel unprepared to handle tasks, duties and other challenges delegated to them. The reason is that they did not acquire the necessary knowledge and skills during student training [7]. International studies conducted in the late 1980s confirmed this deficiency in training, seeing that more than half of the competencies in nursing curricula did not appear in the clinical practice environment [8]. Such limitations could lead to poor quality care in the clinical practice environment. Thus, research addressing these challenges is of utmost importance.

The mentioned challenges are prevalent in developing countries, which typically are affected more by staff shortages, lack of resources and a high burden of disease. Although the unequal allocation of resources entail a worldwide problem, the degree of these inequalities differs depending on the country. In SA, people experience inequality even stronger due to poor availability of healthcare staff across various socio-economic divides: public/private, rural/urban, poor/wealthy, and high public dependent/low medically insured groups [9]. Such inequalities also cause qualified healthcare professionals (HCPs) to immigrate, seeking out countries with improved clinical environments.

Since January 2008, the SA government attempted to address these inequalities in especially rural communities. The main aim was to prevent newly qualified HCPs from leaving the country forthwith by compelling them to do a compulsory year of community service [10]. At that stage, registered nurses (RNs) were not included in the directive. Since 2008, NGRNs in SA who completed their nursing training also had to undertake a compulsory community service year in the public healthcare sector. This service forms part of the requirements of registration as RN with the SA Nursing Council (SANC), the regulatory body of SA nurses, after graduation [11].

All NGRNs who enrolled for the four-year degree or diploma were to be registered with the same specialities as a RN at SANC. However, the nursing degree students’ qualification exit on level 8 of the National Qualification Framework, and diploma students exit on level 7. All nursing education institutions (NEIs) have to follow the standards for nursing education and training as specified by the SANC, to ensure quality training based on an explicated scope of practice [12].

After achieving their undergraduate qualifications, all RNs practise under the SA Nursing Act 33 of 2005, which regulates the nursing profession [12]. In SA, NGRNs are referred to as community service nurses. Community service is described by the SA Nursing Act (No. 33 of 2005) as a SA citizen, who plans to practise a profession for the first time in an arranged category, who must complete paid community service for one year at a public health facility [12].

During the 2011 SA National Nursing Summit, education and training were identified as one of the challenges facing the SA nursing profession. It was agreed that this issue had to be addressed in order to reconstruct and revitalise the SA nursing profession and create “a long and healthy life for all SA” [12, 13]. However, it is a challenge to revitalise the nursing profession. In this regard, research indicated that numerous nurses, including NGRNs, do not have the necessary competencies in various fields of nursing – which may cause poor-quality patient care [13]. Studies add that nurses’ competencies are usually determined by their managers’ evaluations and self-reporting, however, there is insufficient proof that these approaches predict optimal competence [8].

Competencies can be understood as knowledge, skills and attitudes (KSAs) [14] that enable a nurse to make the correct decisions when performing specified tasks in the clinical environment [15]. The SANC defines competencies as the “ability of a practitioner to integrate the professional attributes including, but no limited to knowledge, skills, judgement, values and beliefs (attitudes in this study) required to perform as a RN and midwife in all situations and practice settings” [12].

## Methods

### Aim

The study aimed to develop a competency profile for NGRNs based on the perceptions of both nurse educators and final-year nursing students, regarding the KSAs needed to deliver quality patient care in SA.

### Design

A qualitative descriptive design was followed to describe nurse educators and final-year nursing students’ perceptions in their natural setting.

### Setting

The setting for the study were NEIs which consisted out of one university and two nursing colleges in a single province within South Africa. The NEIs presented either a four-year nursing degree (university) or diploma (college).

### The participants

Forty-two participants were recruited purposely, comprising 23 nurse educators (group 1) and 19 final-year nursing students (group 2) from the NEIs. The demographic profile for both nurse educators and final-year nursing students is shown in Table 1.

**Table 1:** Demographic profile for both nurse educators’ and final-year nursing students (*N* = 43)

Item	Category	Frequency (f)	Percentage (%)
**Nurse educators’ (*n*** **= 23)**			
Age	19–29	2	9%
	30–39	4	17%
	40–49	4	17%
	50–59	12	52%
	60–69	1	5%
Race	Caucasian	11	48%
	African	11	48%
	Mixed race	1	4%
Gender	Male	2	9%
	Female	21	91%
Nursing specialities	Midwifery	7	31%
	General nursing	5	22%
	Psychiatry nursing	1	4%
	Community nursing	3	13%
	Critical care nursing	2	9%
	Operating theatre nursing	1	4%
	Clinical health assessment, treatment and care	3	13%
	Paediatric nursing	1	4%
**Final-year nursing students (*n*** **= 19)**			
Age	19–29	15	79%
	30–39	2	11%
	40–49	1	5%
	50–59	1	5%
Race	Caucasian	6	32%
	African	13	68%
Gender	Male	4	21%
	Female	15	79%

### Data collection

Data collection commenced during 2017. Semi-structured interviews were conducted in English by one of the authors at each of the NEIs and lasted approximately 30 min each. All the interviews were guided by an interview guide (see Table 2).

**Table 2:** Interview guide for nurse educators and final-year nursing students

**Interview guide**	
• What does quality patient care mean to you?	
• Based on your own experiences, what do you think the challenges are that comserve nurse (NGRNs) face when attempting to deliver quality patient care?	
• What do you think must be done by NEIs to overcome these challenges to deliver quality patient care?	
• Do you think the problems which clinical practice environments experience with regard to quality patient care are due to a lack of KSAs, please elaborate?	
• Which of these three concepts knowledge, skills or attitudes do you think is the biggest problem?	
• Do you think the training that nursing students receive at NEIs is adequate to develop the necessary KSAs to deliver quality patient care? Please elaborate.	
• Do you think the nursing curriculum is presented by the NEI in such a way that the focus is on the KSAs which nursing students need to effectively deliver quality patient care when appointed as comserve nurse (NGRN)? Please elaborate.	
• What should the NEI focus on more with regard to KSAs?	
• What does the NEI currently focus on with regard to the KSAs?	
• In your opinion, what specific KSAs do comserve nurses (NGRNs) need to deliver quality patient care?	

### Rigour

Criteria were applied for trustworthiness in qualitative inquiry [16], to enhance the study’s rigour. Credibility was ensured by using purposive sampling to include participants who would provide the richest data necessary to reach the aim of the study. Transferability was ensured by using a representative population to achieve the aim of the study. Dependability was reached by using an interview guide to ensure the same questions were posed to all the participants. Furthermore, the questions in the interview guide were pre-tested with two nursing educators and three final-year nursing students. After the pre-test no changes were necessary on the interview guide as participants understood the questions well and considered it relevant. In order to ensure repeatability in other contexts an effort was made to in detail describe the design and method of the study to the reader. Finally, neutrality was maintained by using an independent transcriber and the mentioned interview guide to ensure the same questions were directed to all the participants.

### Data analysis

Data analysis consisted of two steps. During the first step all the interviews were transcribed verbatim by an independent person. The interviews and transcripts were anonymous and labelled by codes. Both group’ transcripts were analysed separately using thematic analysis [17] based on the three predetermined themes: knowledge, skills and attitudes. The six phases of thematic data analysis are explained subsequently.

Phase 1 entailed familiarisation with the data to obtain an overview. For Phase 2 initial data were generated, predetermined due to the adopted deductive approach. In addition, undetermined themes and sub-themes were developed within each main theme. During phase 3, themes and sub-themes were searched and identified, organised and categorised into smaller and more comparable themes and sub-themes. The result was an overview of how the different codes combine to form an overlapping theme and to identify the relations between the different levels of the themes. In phase 4, all the themes were reviewed to determine similarities within the dataset. During phase 5, all the predetermined themes, themes and sub-themes were defined and refined and the relationships determined to prevent themes from overlapping. Final names were allocated to all sub-themes and sub-sub-themes and the researcher ensured the names reflected the content of the data accurately. Finally, the predetermined themes, themes and sub-themes were collaborated with the co-coder to ensure that it followed a consistent pattern [17].

In step 2 a competency profile was developed by listing the results of the interviews, thereby combining the predetermined themes consisting of more comparable themes and sub-themes of both groups.

## Results

The results are presented based on the 2 steps in the data analysis.

### Step 1

#### Perceptions of nurse educators’ and final-year nursing students

The results of both the nurse educators and final-year nursing students are provided in Table 3. The findings indicated three *predetermined* (knowledge, skills and attitudes), *themes*, *similar sub-themes* between nurse educators and final-year nursing students, *unique sub-themes* of nurse educators and *unique sub-themes* of final-year nursing students with quotations. The results showed various similarities in the themes and sub-themes (see Table 3).

**Table 3:** KSAs needed NGRNs based on the perceptions of both nurse educators’ and final-year nursing students

Predetermined themes	Themes	***Similar*** sub-themes between nurse educators and final-year nursing students	***Unique*** sub-themes of nurse educators	***Unique*** sub-themes of final-year nursing students
**Knowledge**	**Theoretical knowledge**	**Anatomy & physiology**	**Community nursing**	**Microbiology**
		*‘I think basic anatomy, physiology, pharmacology’* (Nurse educator).	*‘Community, where it talks about community’*	*‘Microbiology and biology.’*
		*‘His body from the inside out.’* (Student)		
		**Pharmacology**	**Patient safety**	**Management & administration**
		*‘I think basic anatomy, physiology, pharmacology’* (Nurse educator).	*‘Always make sure that you work safe, protect yourself, protect your patients.’*	*‘Everything that comes under Human Resources.’*
		*‘Pharmacology, it’s a subject that teaches you about all the different types of drugs and how they work.’ *(Student)		
		**Midwifery**	**Biochemistry**	**Research**
		*‘Able to deliver a baby.’* (Nurse educator)	*‘Biochemistry is important.’*	*‘Many of them don’t keep up to date with new things coming out, new research.’*
		*‘Foundation about maternity.’* (Student)		
		**Sociology**	**Pathology**	
		*‘Sociology specific to us.’* (Nurse educator)	*‘All the conditions, the pathology.’*	
		*‘Community we have sociology’*. (Student).		
		**Psychiatry**	**Intensive care**	**Follow-up of patients**
		*‘Third level and fourth level with the psychiatric.’* (Nurse educator)	*‘Fourth year, they … things like ICU.’*	*‘Do follow-ups if needed.’*
		*‘We also do psychiatry for a year’* (Student)		
		**Ethos of care**	**Clinical manifestations**	
		*‘Teach them the ethics, to link in with their knowledge and skills, and how to work with the patients.’* (Nurse educator)	*‘The conditions, the pathology, the clinical manifestations.’*	
		*‘Ethos, that teaches you on how to present yourself.’* (Student)		
		**Medication administering procedures and effects**	**Fundamental nursing**	
		*‘Know the effect of medication on the body’* (Nurse educator)	*‘I think the knowledge they need to have all the basic skills.’*	
		*‘Know which medication helps for hypertension, these are the side effects and why do you use it.’* (Student)		
		**Nursing diagnosis**	**Patient diagnosis**	
		*‘They have to be able to do basic diagnosis and treatment’* (Nurse educator)	*‘They have to be able to do basic diagnosis and treatment.’*	
		*‘Basic nursing skills, you need to know in order to diagnose.’* (Student)		
		**Diseases**	**Patient evaluation**	
		*‘Knowledge of the impact of the prominent diseases and ailments.’* (Nurse educator)	*‘They need to know how to evaluate a patient.’*	
		*‘Let’s say a specific condition or a disease then immediately when that person presents then you would know.’* (Student)		
		**Patient assessment**	**Patient needs**	
		*‘The independent (NQRN) must know how to do the assessment of that patient.’* (Nurse educator)	*‘Knowledge of the person the needs of the patient.’*	
		*‘To deliver quality care to the patient you need to know how to assess.’* (Student)		
		**Patient treatment**	**Ward and hospital functioning**	
		*‘Know how to issue treatment according to the illness of the patient.’* (Nurse educator)	*‘They need to know how a ward functions and how a hospital functions.’*	
		*‘Know how to issue treatment according to the illness of the patient.’* (Student)		
		**Patient referrals**	**Infection control**	
		*‘Should refer when it is necessary to refer that particular patient.’* (Nurse educator)	*‘Infection control, it’s very important.’*	
		*‘So that you know this one (patient) I have to refer.’* (Student)		
		**Community profile**		
		*‘Knowledge of the diseases and the profile and area that they are working in.’* (Nurse educator)		
		*‘Especially the community, they don’t know much about the conditions that are around’* (Student)		
	**Holistic care**	**Physical**	**Social**	
		*Quality patient care means caring for a patient in totality, which means you care for his or her physical wellbeing* (Nurse educator).	*‘Quality patient care means taking total care of the patients’ needs, the physicals, socials.’*	
		*‘Taking care of patient in every needs of the patient, physically.’* (Student)		
		**Spiritual**		
		*‘It is if you nurse a patient holistically, spiritually.’ (Nurse educator)*		
		*‘Quality patient care, its nursing a patient like holy, spiritually.*’ (Student)		
		**Psychological**		
		*‘This includes the physical, psychological and spiritual.’* (Nurse educator)		
		*‘Not just looking at his physical but also his psychological.’* (Student)		
		**Emotional**		
		*‘Attended in all areas of his being, not only physical but also spiritual and emotional.’* (Nurse educator) *‘*		
		*It is to holistically care for the patient. You must care physically and emotionally.’* (Student)		
	**Cultural diversity**	**Multilingual**	**Inclusiveness**	**Acceptance of cultural diversity**
		*‘Also must try to be multicultural in terms of language.’* (Nurse educator)	*‘You have to accept people regarding their culture, beliefs, race and sexuality.’*	*‘Their cultures differently, know how to deal with it, and the likes and dislikes of the patient.’*
		*‘Improve English, for most of the nurses.’* (Student)		
	**Code of conduct**	**Professionalism (image)**		**Professionalism (attitude)**
		*‘Now you see nurses in theatre wearing rings, wearing these fake nails, wearing.’* (Nurse educator)		*‘And your professional conduct.’*
		*‘On how to handle the patients and how to present yourself.’* (Student)		
		**Policies and procedures**		**Scope of practice**
		*‘If a nurse knows what independent functions are, which are the practice of functions that a person do within realize of the law or regulations.’* (Nurse educator) *‘I think if you can know the policies, code of conduct.’* (Student)		*‘I think if you can know the policies, code of conduct, scope of practice.’*
				**Nurses’ rights**
				*‘Their rights, [NGRNs] need to know their rights.’*
				**Confidentiality and privacy**
				*‘Towards my patients I must always treat them with dignity and respect and ensure privacy at all times and confidentiality.’*
**Skills**	**Interpersonal skills**	**Conflict management**	**Emotional intelligence**	**Interaction skills**
		*‘Able to manage conflict.’* (Nurse educator)	*‘I think she requires emotional intelligence.’*	*‘I think definitely interaction between people.’*
		*‘I would say, both communication skills and conflict skills.*’ (Student)		
		**Communication**	**Accommodating**	**Patient relationships**
		*‘You also need your interpersonal skills, like your communication.’* (Nurse educator)	*‘Working together, accommodating each other.’*	*‘You would have to build a relationship with your patient.’*
		*‘I think the communication skills are very important* (Student)		
		**Listening**	**Advocate for a patient**	**Calm**
		*‘Really being able to listen to your patient, hear what they are saying.’* (Nurse educator)	*‘We must advocate for the patient.’*	*‘When the patient is angry you must always be calm.’*
		*You need to listen to what the patient is saying here.’* (Student)		**Courtesy**
				*‘You must treat everybody with courtesy.’*
		***Respect***		
		*‘Respect humanity. It doesn’t matter their social class.’* (Nurse educator)		
		*‘Respect in general if you can respect a patient then respect the others then you can provide the good patient care.’* (Student)		
		**Team work**		
		*‘They must be able to cooperate within the team that they are working in, because if they don’t have that, then they don’t work together.’* (Nurse educator)		
		‘*You are going to be working in a team.’* (Student)		
	**Management skills**	**Leadership**	**Business skills**	**Administration**
		*‘Management skill or leadership skill she has to display.’* (Nurse educator)	*‘Will also have business skill.’*	*‘The admin of a clinic or a hospital.’*
		*‘You must be a leader; you must know how to lead.’* (Student)		
		**Management of a ward**	**Time management**	**Computer skills**
		*‘And then another thing, for example, we, we should teach them how to run a ward.’*(Nurse educator)	*‘Specifically time management skills.’*	*‘You need to have computer skills, there are a lot of computer in it.’*
		*‘You must know how to manage the ward and to schedule time off.’* (Student)		
	**Administrative skills**		**Record keeping**	
			*‘Yes, record keeping is also very important.*’	
			**Report writing**	
			*‘They need to have good writing skills, clear writing and good report.’*	
	**Practical skills**	**Insertion of intravenous drip**	**Performance of general nursing procedures**	**Cardio-pulmonary resuscitation**
		*‘Insertion of the intravenous drip.’* (Nurse Educator)	*‘They should be able to perform different procedures.’*	*‘There is a procedure of CPR and then they say practice on a doll.’*
		*‘Able to put a drip* (Student)		
		**Administering of an injection**	**Immunisations**	**Management of aggressive patients**
		*‘To give an injection on the buttocks, on the arm.’* (Nurse educator)	*‘To give an immunisation.’*	*‘If a patient becomes aggressive you must be able to handle it.’*
		*‘You (NQRN) are going to give injections, so that is basic things that you must know and be able to do.’* (Student)		
		**Delivering a baby**	**Administering of medication**	**Bed-wash of a patient**
		*‘Specialized knowledge also of delivering babies.’* (Nurse educator)	*‘Medication administration skills.’*	*‘How to bed-wash a patient.’*
		*‘Experience to know how to deliver a baby.’* (Student)		
		**Monitoring vital signs**	**Antenatal care**	
		*‘How to do blood pressure’* (Nurse educator)	*‘Or antenatal care actually.’*	
		*‘How to monitor the vital signs.’* (Student)		
		**Blood drawing**	**Glucose testing**	
		*‘Putting drips and draw bloods’* (Nurse educator)	*‘How to bath a patient, test glucose.’*	
		*‘Draw blood, know how to do it.’* (Student)		
			**Patient observation**	
			*‘They should be able to observe. If you*	
			*see something on a patient then you should be able to do something with that.’*	
	**Personal skills**	**Independence**	**Creative thinking**	**Flexibility**
		*‘Do everything on their own.’* (Nurse educator)	*‘I would say ability to think out of the box.’*	*‘Must be flexible as well.’*
		*‘Be able to work independently.’* (Student)		
		**Adaptability**	**Emotional stability**	**Diligent**
		*‘They need to adapt to their circumstances*.’ (Nurse educator)	*‘Need to have emotional stability herself in order to be able to go on.’*	*‘Don’t do anything half measure – do the procedures properly and don’t be lazy.’*
		*‘You must be adaptable.’* (Student)		
		**Patience**	**Coping**	**Resilient**
		*‘Patience I think. Patience.’* (Nurse educator)	*‘Be psychologically prepared.’*	*‘Must try to keep their head above water and they would not be able to go sit.****’***
		*‘When it comes to patients we should try to have more patience.’* (Student)		
		**Assertive**	**Integrity**	
		*‘Assertiveness and certain stubbornness in sticking to do what is right.’* (Nurse educator)	*‘Be responsible and trustworthy so even if no one is there to see what you do.’*	
		*‘Be more assertive.’* (Student)		
		**Responsible and trustworthy**	**Confidence**	
		*‘Really to be responsible and trustworthy so even if no one is there to see what you do.’* (Nurse educator)	*‘Have the attitude of I can do this, I am able, I have all the competency.’*	
		*‘Only thing that we have to understand to know more is that is to be responsible.’* (Student)		
		**Courageous**	**Endurance**	
		*Courageous enough, you know, if he can carry out quality nursing care.’* (Nurse educator) *‘Show that courage.’* (Student)	*‘They must have endurance because it’s not easy where they go.’*	
		**Problem-solving**	**Determination**	
		*‘Problem solving skills, it’s very much important.’* (Nurse educator)	*‘They also need determination.’*	
			**Values**	
		*‘Your problem solving abilities would follow logically.’* (Student)	*‘They must have values.’*	
		**Ability to prioritise**		
		*‘The skill to prioritise.’* (Nurse educator)		
		*‘Be able to prioritise and to say this is what must be done now, the next task is this one.’* (Student)		
		**Critical analytical thinking**		
		*‘They need that critical analytical way of thinking.’* (Nurse educator)		
		*‘It must be a critical thinker.’* (Student)		
		**Decision-making**		
		‘*The skill to make decision, decision-making skills.’* (Nurse educator)		
		*‘Independent in terms of making decisions.’*(Student)		
		**Crisis management**		
		*‘They have to have effective crises management.’* (Nurse educator)		
		*‘Handle an emergency situation.’* (Student)		
		**Innovative thinking**		
		*‘So they would learn to think in innovative ways to do something.’* (Nurse educator)		
		*‘You must think on your feet.’* (Student)		
		**Improvise**		
		*‘They must really think and what they can do if there are no resources.’* (Nurse educator		
		*‘If we are learning a particular skill, they will tell us okay but you can use this or that as an alternative.’* (Student)		
**Predetermined themes**		***Similar*themes between nurse educators and final-year nursing students**	***Unique*themes of nurse educators**	***Unique*themes of final-year nursing students**
**Attitudes**		**Positive**	**Compassionate**	**Non-judgemental**
		*‘You should have a positive attitude with the patient.’* (Nurse educator)	*‘She needs as well to have compassion.’*	*‘Like a teenage girl who needs to go for family planning.’*
		‘You have to have a positive attitude if you are going to work.’ (Student)		
		**Caring**	**Passion**	
		*‘If you want to be a nurse, you must have a caring attitude.’* (Nurse educator)	*‘Be passionate about what you are doing.’*	
		*‘You want to help patient to get well and you want to show love to patients.’* (Student)		
		**Humble**	**Approachable**	
		*‘To place their own needs aside so that the patient always comes first.’* (Nurse educator)	*‘Approachability, welcoming and that reassurance and all those.’*	
		*‘You are never too good to do things like bed wash or changes bed pans or a patient’s diaper.’* (Student)		
		**Friendly**	**Sensitive**	
		*‘Friendliness knowing that the patient comes first.’* (Nurse educator)	*‘Need to be sensitive.’*	
		*‘Friendly towards the patient, the personnel.’* (Student)		
		**Empathetic**	**Helpful**	
		*‘The nurse should show empathy, understanding of the patients stories.’* (Nurse educator)	*Think of how best can I help this patient.’*	
		*‘The nurse should show empathy, understanding of the patients.’* (Student)		
		**Life-long learning**		
		*‘You must really be open to learn don’t think I know everything because I am qualified, and realize that you will be a life-long learner.’* (Nurse educator)		
		*‘You would have to learn from the other nurses.’* (Student)		
		**Going the extra mile**		
		‘*And also you must go an extra mile.’* (Nurse educator) *‘Nursing is about giving but you get very little back.’* (Student)		

#### Knowledge

In the predetermined theme, *knowledge,* the following themes emerged: theoretical knowledge, holistic care, cultural diversity and code of conduct, together with their various sub-themes.

In the theme *theoretical knowledge,* similar sub-themes emerged between the nurse educators and final-year nursing students, namely: anatomy & physiology, pharmacology, midwifery, sociology, psychiatry and ethos of care, medication administering procedures and effects, nursing diagnosis, diseases, patient assessment, patient treatment, patient referrals and community profile. However, the nurse educators specifically added specific sub-themes: community nursing, patient safety, biochemistry, pathology and intensive care, clinical manifestations, fundamental nursing, patient diagnosis, patient evaluation, patient needs, ward and hospital functioning and infection control. In turn, the final-year nursing students highlighted the following as important aspects of theoretical knowledge that NGRNs should acquire: microbiology, management & administration, research, and follow-up of patients.

The sub-theme *holistic care* was mentioned by both nurse educators and final-year nursing students which included aspects of physical, spiritual, psychological and emotional care, whereas only the nurse educators pointed out social care.

*Cultural diversity* was also a sub-theme emerging together with being multilingual mentioned by both groups. In this regard, the nurse educators uniquely added inclusiveness, whereas and the final-year nursing students also included acceptance of cultural diversity.

In the theme *code of conduct,* both educators and students mentioned professionalism, particularly concerning the image of the nurse as well as policies and procedures. In addition the final-year nursing students also highlighted professionalism, especially attitudes, the scope of practice, nurses’ rights as well as confidentiality and privacy.

#### Skills

As part of the second predetermined theme, *skills,* the following themes emerged: interpersonal, management, administrative, practical and personal skills, with respective sub-themes.

*Interpersonal skills* were considered equally important to both groups, were conflict management, communication, listening, respect and team work. In this regard, the nurse educators uniquely added emotional intelligence, being accommodating and being an advocate for a patient. The final-year students specifically added interaction skills, patient relationships, being calm and courtesy.

In the theme *management skills,* both groups included leadership and management of a ward. The nurse educators added business skills and time management, whereas final-year students added administration and computer skills.

In addition to the theme *administrative skills*, only the nurse educators mentioned record keeping and report writing.

In the theme *practical skills,* both groups included insertion of intravenous drip, administering of an injection, delivering a baby, monitoring vital signs and blood drawing. The nurse educators uniquely added performance of general nursing procedures, immunisations, administering of medication, antenatal care, glucose testing and patient observation, whereas the final-year nursing students uniquely added cardio-pulmonary resuscitation, management of aggressive patients and bed-wash of a patient.

For both groups, the theme *personal skills* captured the following sub-themes: independence, adaptability, patience, being assertive, responsible and trustworthy and courageous, problem-solving, ability to prioritise, critical analytical thinking, decision-making, crisis management, innovative thinking and improvise. The nurse educators uniquely added the following aspects: creative thinking, emotional stability, coping, integrity, confidence, endurance, determination and values. From their side, the final-year students’ added flexibility, being diligent and resilient.

#### Attitudes

*Attitudes* included the following themes by both groups: positive, caring, humble, friendly, empathetic, life-long learning and going the extra mile. As additional themes, the nurse educators mentioned being compassionate, having passion and being approachable, sensitive and helpful. The final-year students added the theme being non-judgemental.

### Step 2

#### Competency profile for NGRNs based on the perceptions of nurse educators’ and final-year nursing students

Competency refers to the KSAs, values and abilities that employees must apply to meet the standard work performance levels of the organisation. Profile refers to an outline or a structure that is unique and recognisable [18]. The competency profile describes structured competencies by combining the predetermined themes, themes and sub-themes from step 1 (see Table 4).

**Table 4:** Competency profile for NGRNs (Authors’ own work)

**Knowledge**		
**Theoretical knowledge**	• Anatomy & physiology • Pharmacology • Midwifery • Sociology • Psychiatry • Ethos of care • Medication administering procedures and effects • Nursing diagnosis • Diseases • Patient assessment • Patient treatment • Patient referrals • Community profile • Community nursing • Patient safety	• Biochemistry • Pathology • Intensive care • Clinical manifestations • Fundamental nursing • Patient diagnosis • Patient evaluation • Patient needs • Ward and hospital functioning • Infection control • Microbiology • Management & administration • Research • Follow-up of patients
**Holistic care**	• Physical • Spiritual • Psychological	• Emotional • Social
**Cultural diversity**	• Multilingual • Inclusiveness	• Acceptance of cultural diversity
**Code of conduct**	• Professionalism (image & attitude) • Policies and procedures • Scope of practice	• Nurses’ rights • Confidentiality and privacy
**Skills**		
**Interpersonal skills**	• Conflict management • Communication • Listening • Respect • Team work • Emotional intelligence	• Accommodating • Advocate for a patient • Interaction skills • Patient relationships • Calm • Courtesy
**Management skills**	• Leadership • Management of a ward • Business skills	• Time management • Administration • Computer skills
**Administrative skills**	• Record keeping	• Report writing
**Practical skills**	• Insertion of intravenous drip • Administering of an injection • Delivering a baby • Monitoring vital signs • Blood drawing • Performance of general nursing procedures • Immunisations • Administering of medication	• Antenatal care • Glucose testing • Patient observation • Cardio-pulmonary resuscitation • Management of aggressive patients • Bed-wash of a patient
**Personal skills**	• Independence • Adaptability • Patience • Assertive • Responsible and trustworthy • Courageous • Problem-solving • Ability to prioritise • Critical analytical thinking • Decision-making • Crisis management • Innovative thinking	• Improvise • Creative thinking • Emotional stability • Coping • Integrity • Confidence • Endurance • Determination • Values • Flexibility • Diligent • Resilient
**Attitudes**		
	• Positive • Caring • Humble • Friendly • Empathetic • Life-long learning • Going the extra mile	• Compassionate • Passion • Approachable • Sensitive • Helpful • Non-judgemental

## Discussion

NGRNs face several challenges when entering the practice environment after completing their studies. Typical challenges are lack of independence, low confidence and feelings of incompetence [3, 4], thus, necessitating further investigation into the competencies nurses require to deliver quality patient care. As far as the authors could establish this was the first competency profile developed based on the perceptions of both nurse educators and final-year nursing students who were HCPs working within the clinical environment. Therefore, such a profile can help NGRNs with challenges and their transition into the clinical environment.

The challenges NGRNs face during their transition into the clinical environment are compounded by rapidly growing and more complex healthcare systems. In such a context a high level of competence is expected of HCPs, especially RNs [19, 20], which include NGRNs who perform their compulsory community service year.

As the case is worldwide, the professional body for nursing in SA, the SANC, typically have established acts, regulations and procedures for nursing training to ensure the necessary competencies are acquired during their four years of training. However, the expectations and challenges of the clinical environment at times vary due to several factors. NGRNs have to contend with staff shortages, lack of resources, the high burden of disease and the large number of people who depend on the public healthcare sector for medical assistance.

The proposed competency profile highlights the nurse educators’ and final-year nursing students’ perceptions about competencies that NGRNs potentially need after completing their studies, in order to deliver quality patient care when entering the clinical practice environment. Literature confirms that nurses with the necessary competencies render high-quality services to patients [21]. Competence is a combination of the “complex attributes of KSAs” [15], which help nurses make the correct decisions when performing in particular circumstances. Several scholars add that competency is not that evident when observing individuals’ behaviour, but is determined by their performance, when undertaking tasks. Consequently, competence can be explained as an individual’s “cognitive approach to a task, encompassing the multiple attributes of KSAs” [15].

At times the required level of competence expected of NGRNs remains under defined [22]. In addition, NGRNs feel incompetent when performing a range of clinical procedures, which should have been developed during their undergraduate programmes in various clinical environments [22]. In the Americas, the ‘Quality and Safety Education for Nurses’ with the assistance of the National Advisory Board, attempted to address this deficiency by developing competencies that enhance quality and safety in patient care. Such competencies could serve as guidelines when developing curricula for academic programmes focusing on the transition to clinical practice environments and other post-basic forms of learning. A number of these competencies were also highlighted in the findings of the present study. These competencies include patient-centred care (holistic care in this study), teamwork and partnerships, enhancement of quality, informatics and security.

Finland also identified eight competence areas for nursing students, namely: “professional/ethical values and practice, nursing skills and intervention, communication and interpersonal skills, knowledge and cognitive ability, assessment and improvement of quality in nursing, professional development, leadership, management and teamwork and research utilization” [19]. These areas were also found in the present study. In addition to these competencies, the present study identified other unique aspects in the theme. Knowledge such as cultural diversity consisting of multi-lingualism, inclusiveness and accepting the cultural diversity.

In the findings of this research study, knowledge competencies included theoretical knowledge, holistic care and code of conduct, which are all relatively common matters to be knowledgeable about. However, an additional competency, namely cultural diversity, was identified within the SA context and indicated the importance of contextualising aspects to various settings to help ensure quality patient care, especially in countries with diverse ethnic groups such as SA. It is therefore not surprising that knowledge about various cultural diversities and being multi-lingual was highlighted as essential for NGRNs.

The high demand for healthcare services by patients with multifaceted needs requires more highly skilled employees [19, 20], especially RNs. Such required professionalism applies especially to developing countries where the largest part of the population depends on the public health sector, which typically is fraught with staff shortages, high workloads, insufficient resources and inadequate infrastructure [9, 23]. Interpersonal relationships, management and practical skills are also important for nurses seeking to deliver quality care [20]. In addition, the present study pointed out the importance of administrative and personal skills. Seemingly, these mentioned skills are obvious competencies that all nurses should possess in the public healthcare sector, except for business and IT skills. However, when considering the rapidly growing and more complex healthcare systems, the addition of the last two mentioned skills is highly relevant.

A less ostensive yet expected skill is attitude, which can be defined as a “lasting belief, feeling and tendency to behave in a certain way towards a specific person, object, idea or issue” [18]. It is extremely important that nurses portray a benign attitude towards patients. In response, patients can build a trusting relationship, feel comfortable to discuss private problems and allow physical examinations performed on them. On the other hand, the seemingly uncaring attitude of certain nurses is not elicited purposefully. Especially in the SA public healthcare sector, salient factors frustrate nurses, for example, staff shortages, overcrowding, lack of resources and workloads. Such frustrations may cause nurses to exhibit an uncaring attitude [23]. The study explored different attitudes such as being positive, caring, humble and friendly. Such guidelines may help NGRNs understand which specific attitudes may be appropriate to deliver quality care to patients.

## Conclusions

NGRNs are just as central to the delivery of quality patient care as other RNs. However, quality care may be hampered when NGRNs make the transition from student to RN. This study investigated the perceptions of nurse educators and final-year nursing students about the KSAs they felt were necessary for NGRNs to deliver quality care more effectively when entering the practice environment. The perceptions about the KSAs led to the development of a competency profile for NGRNs. This competency profile is not compared with the currently approved nursing curriculum of SA and does not suggest the redevelopment thereof. However, it does provide some indication of the KSAs that prospective NGRNs may need to enable them to deliver quality patient care after successful completion of their studies. NEIs may therefore use this competency profile to determine where they may place more emphasis during nursing training to assist these nurses to deliver quality care in their future role as NGRN. Nursing students may also be made aware of these KSA’s in order for them to know where they should focus more attention to during their studies. The proposed competency profile could also reduce complaints from colleagues and patients, lower disciplinary actions taken against NGRNs, decrease the need for re-training and improve the practice environments’ overall image.

### Limitations of the study

The present study focused on nurse educators and final-year nursing students from a single province in SA. Therefore, future studies could consider incorporating other HCPs in the practice environment, including patients, as both are in direct contact with NGRNs and may therefore provide relevant information to expand on the present findings.

Furthermore, this study explored only the perceptions of the nurse educators and final-year nursing students about KSAs, based on individual practical experience. Future studies could consider including nursing curricula or job descriptions in such an analysis.

## Data Availability

All analysed data of the study are included in this published article.

## Abbreviations

HCPs: Healthcare professionals
KSAs: Knowledge, skills and attitudes
NEIs: Nursing Education Institutions
NGRNs: Newly graduated registered nurses
RNs: Registered Nurses
SA: South Africa
SANC: South African Nursing Council
